# The genome sequence of the Vagrant Hoverfly, *Eupeodes corolla*e (Fabricius, 1794)

**DOI:** 10.12688/wellcomeopenres.19099.1

**Published:** 2023-03-08

**Authors:** Duncan Sivell, Olga Sivell, Natural History Museum Genome Acquisition Lab, Darwin Tree of Life Barcoding collective, Wellcome Sanger Institute Tree of Life programme, Wellcome Sanger Institute Scientific Operations: DNA Pipelines collective, Tree of Life Core Informatics collective, Will L. Hawkes, Karl R. Wotton, Darwin Tree of Life Consortium

**Affiliations:** 1 Natural History Museum, London, UK; 2 Centre for Ecology and Conservation, University of Exeter, Penryn Campus, Penryn, UK

**Keywords:** Eupeodes corollae, Vagrant Hoverfly, genome sequence, chromosomal, Diptera

## Abstract

We present a genome assembly from an individual female *Eupeodes corollae* (the Vagrant Hoverfly; Arthropoda; Insecta; Diptera; Syrphidae). The genome sequence is 648.2 megabases in span. Most of the assembly is scaffolded into four chromosomal pseudomolecules, including with the X sex chromosome. The mitochondrial genome has also been assembled and is 18.3 kilobases in length.

## Species taxonomy

Eukaryota; Metazoa; Ecdysozoa; Arthropoda; Hexapoda; Insecta; Pterygota; Neoptera; Endopterygota; Diptera; Brachycera; Muscomorpha; Syrphoidea; Syrphidae; Syrphinae; Syrphini; Eupeodes; *Eupeodes*; *Eupeodes corollae* (Fabricius, 1794) (NCBI:txid290404).

## Background

The Vagrant or Migrant Hoverfly, *Eupeodes corollae,* is a common and widespread, medium-sized hoverfly (wing length 5–8 mm) with a long flight season. This hoverfly occurs throughout Britain and can be found in a variety of open or boundary habitats in urban and rural settings, such as parks, gardens, brownfield sites, agricultural land, woodland edge and road verges ( Ball *et al.*, 2011; Ball & Morris, 2015; Stubbs & Falk, 2002). Adults can be seen from March to November and visit a variety of flowers but are usually most frequent in July and August when they can be common on umbellifers ( Ball *et al.*, 2011; Morris, 1998).

Like many Syrphinae hoverflies, *E. corollae* has yellow markings on a dark abdomen, but in this species the pattern is both variable and sexually dimorphic. In males the yellow markings are larger and more square-shaped, whereas in female they are smaller and narrower. The extent to which these yellow markings reach the lateral edge of the abdomen and the colour of setae along this edge are important characters for identification. The male genital capsule of *E. corollae* is much larger than other *Eupeodes* species which is another useful identification feature ( Ball & Morris, 2015; Stubbs & Falk, 2002).

The species is found across Europe, Asia, and North Africa and, as the common name suggests, is a long-distance migrant. During the springtime, the hoverfly has been documented flying at least 105 km across open ocean from the Middle East to Cyprus ( Hawkes *et al.*, 2022), and has been recorded heading south in the autumn through the Alps and the Pyrenees ( Aubert *et al.,* 1976; Williams *et al.*, 1956). In addition, entomological radar studies above southern Britain have confirmed seasonally directed movements, revealed their huge abundance and investigated their responses to wind currents ( Gao *et al.*, 2020; Wotton *et al.*, 2019).

*Eupeodes corollae* has been widely investigated as a predator of aphid crop pests or as a dual-purpose pollinator and biocontrol agent ( Lillo *et al.,* 2021; van Oystaeyen *et al.*, 2022). In southern Britain, 1 to 4 trillion aphids a year are consumed by the offspring of *E. corollae* and the marmalade hoverfly *Episyrphus balteatus*, following their mass arrivals, making this species an important biocontrol agent in agricultural ecosystems ( Wotton *et al.*, 2019). Reflecting this role, E. corollae is commercially available for the biocontrol of aphids.

This species is also one of the few Syrphids with existing genomic resources, including a previous genome assembly, a transcriptomic analysis of chemosensory genes from the antennae, a genome-wide analysis of population genetic structure and has had mutant lines created for the investigation of the molecular basis of aphid detection ( Liu *et al.*, 2019; Wang *et al.,* 2017; Wang *et al.*, 2022; Yuan *et al.*, 2022). The production of a high quality *Eupeodes corollae genome* described here, generated as part of the Darwin Tree of Life project, will further aid in understanding of the biology and ecology of this hoverfly.

### Genome sequence report

**Figure 1.**
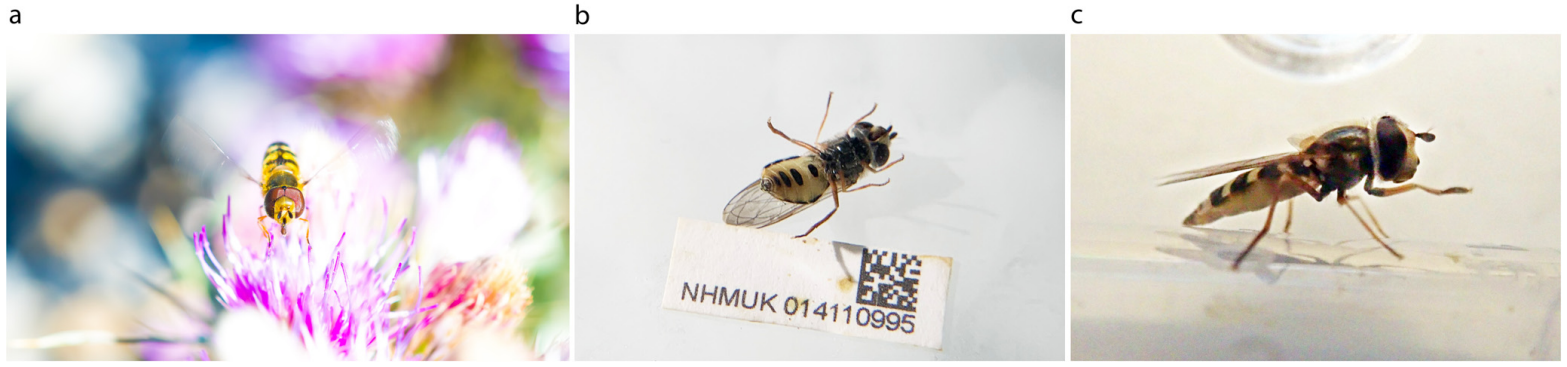
**a**. Photograph of live *Eupeodes corollae* by Will Hawkes. **b**. Ventral view of specimen idEupCoro1, **c**. Lateral view of idEupCoro1.

The genome was sequenced from one female *E. corollae* specimen ( Figure 1 b and c) collected from Luton, UK (latitude 51.89, longitude –0.39). A total of 34-fold coverage in Pacific Biosciences single-molecule HiFi long reads and 34-fold coverage in 10X Genomics read clouds were generated. Primary assembly contigs were scaffolded with chromosome conformation Hi-C data. Manual assembly curation corrected 670 missing or mis-joins and removed 33 haplotypic duplications, reducing the assembly length by 1.53% and the scaffold number by 36.24%, and increasing the scaffold N50 by 6.66%.

**Figure 2.**
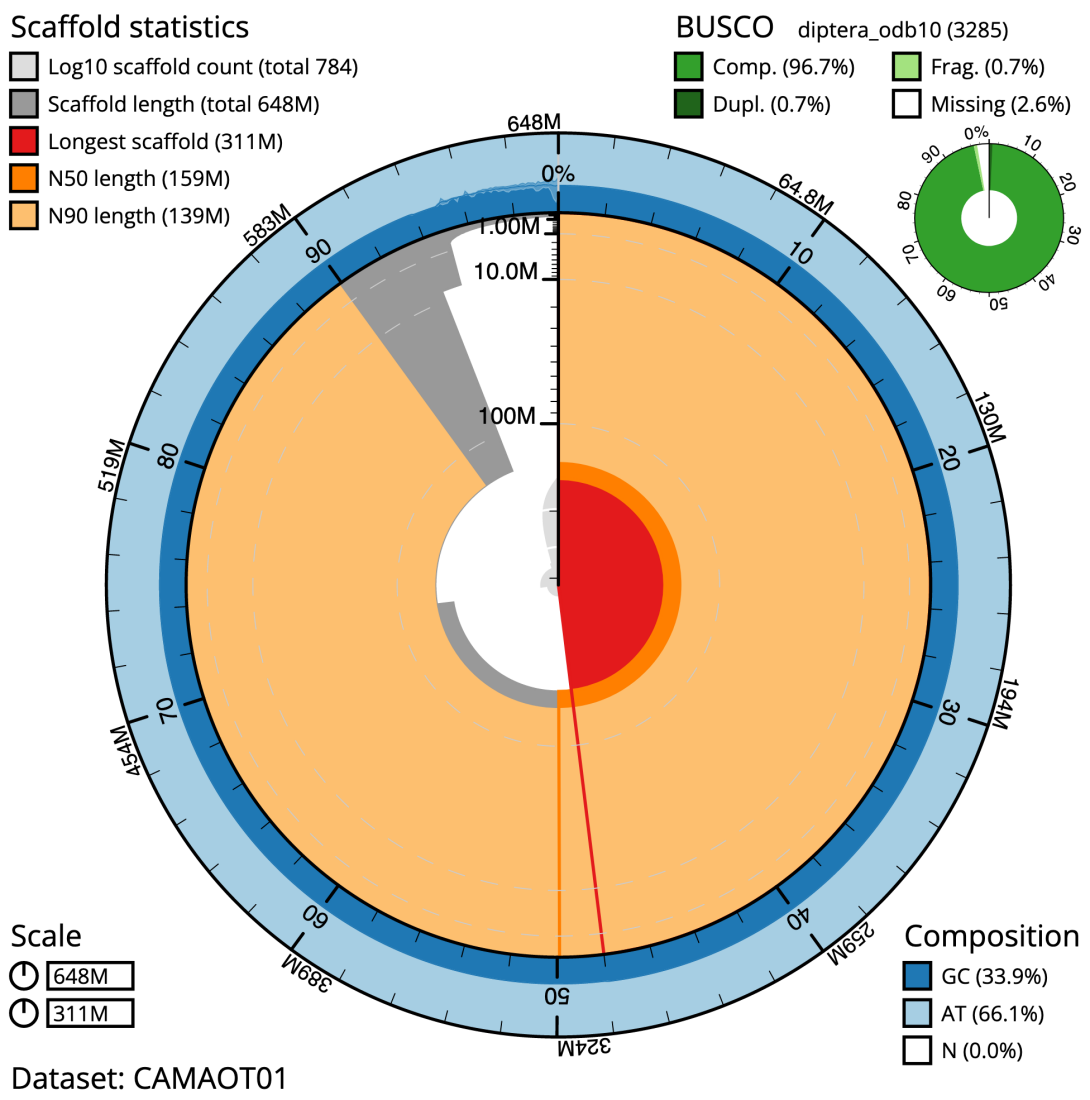
Genome assembly of *Eupeodes corollae,* idEupCoro1.1 *:* metrics. The BlobToolKit Snailplot shows N50 metrics and BUSCO gene completeness. The main plot is divided into 1,000 size-ordered bins around the circumference with each bin representing 0.1% of the 648,213,917 bp assembly. The distribution of scaffold lengths is shown in dark grey with the plot radius scaled to the longest scaffold present in the assembly (311,186,714 bp, shown in red). Orange and pale-orange arcs show the N50 and N90 scaffold lengths (158,619,773 and 139,491,442 bp), respectively. The pale grey spiral shows the cumulative scaffold count on a log scale with white scale lines showing successive orders of magnitude. The blue and pale-blue area around the outside of the plot shows the distribution of GC, AT and N percentages in the same bins as the inner plot. A summary of complete, fragmented, duplicated and missing BUSCO genes in the diptera_odb10 set is shown in the top right. An interactive version of this figure is available at https://blobtoolkit.genomehubs.org/view/idEupCoro1.1/dataset/CAMAOT01/snail.

The final assembly has a total length of 648.2 Mb in 783 sequence scaffolds with a scaffold N50 of 158.6 Mb ( Table 1). Most (95.29%) of the assembly sequence was assigned to four chromosomal-level scaffolds, representing three autosomes and the X sex chromosome. Chromosome-scale scaffolds confirmed by the Hi-C data are named in order of size ( Figure 2– Figure 5; Table 2). The assembly has a BUSCO v5.3.2 ( Manni *et al.*, 2021) completeness of 96.7% (single 96.0%, duplicated 0.7%) using the diptera_odb10 reference set. While not fully phased, the assembly deposited is of one haplotype. Contigs corresponding to the second haplotype have also been deposited.

**Table 1.** Genome data for *Eupeodes corollae,* idEupCoro1.1.

Project accession data		
Assembly identifier	idEupCoro1.1	
Species	*Eupeodes corollae*	
Specimen	idEupCoro1	
NCBI taxonomy ID	290404	
BioProject	PRJEB53926	
BioSample ID	SAMEA7524255	
Isolate information	female: idEupCoro1 (PacBio, Chromium) male: idEupCoro2 (Hi-C)	
Assembly metrics *		*Benchmark*
Consensus quality (QV)	58	*≥ 50*
*k*-mer completeness	100%	*≥ 95%*
BUSCO **	C:96.7%[S:96.0%,D:0.7%], F:0.7%,M:2.6%,n:3,285	*C ≥ 95%*
Percentage of assembly mapped to chromosomes	95.29%	*≥ 95%*
Sex chromosomes	X chromosome	*localised homologous pairs*
Organelles	Mitochondrial genome assembled	*complete single alleles*
Raw data accessions		
PacificBiosciences SEQUEL II	ERR9913036	
10X Genomics Illumina	ERR9904175–ERR9904178	
Hi-C Illumina	ERR9904179, ERR9904180, ERR9904181	
Genome assembly		
Assembly accession	GCA_945859685.1	
*Accession of alternate haplotype*	GCA_945859775.1	
Span (Mb)	648.2	
Number of contigs	2,107	
Contig N50 length (Mb)	2.3	
Number of scaffolds	783	
Scaffold N50 length (Mb)	158.6	
Longest scaffold (Mb)	311.2	

* Assembly metric benchmarks are adapted from column VGP-2020 of “Table 1: Proposed standards and metrics for defining genome assembly quality” from ( Rhie *et al.*, 2021). ** BUSCO scores based on the diptera_odb10 BUSCO set using v5.3.2. C = complete [S = single copy, D = duplicated], F = fragmented, M = missing, n = number of orthologues in comparison. A full set of BUSCO scores is available at https://blobtoolkit.genomehubs.org/view/Eupeodes%20corollae/dataset/CAMAOT01/busco.

**Figure 3.**
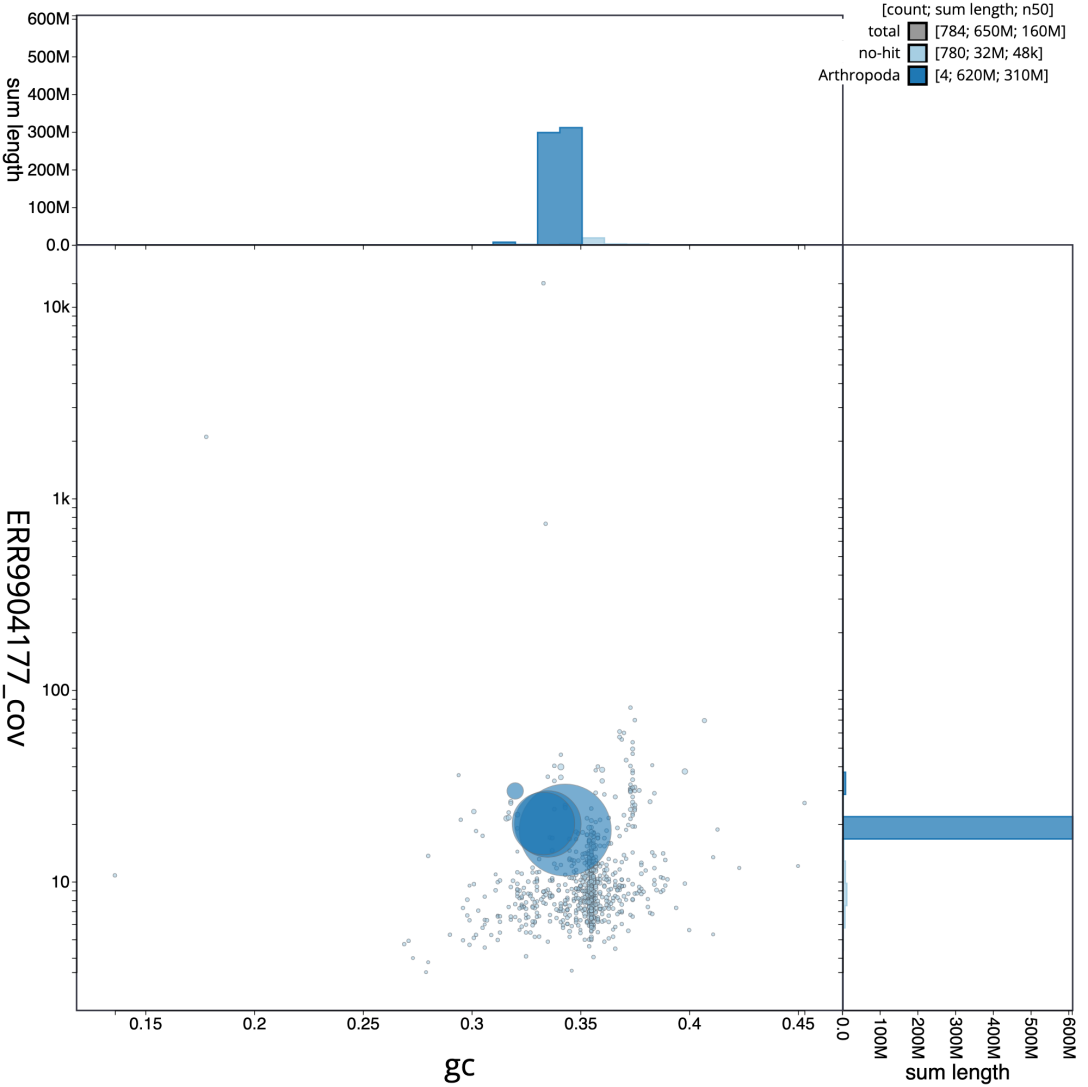
Genome assembly of *Eupeodes corollae,* idEupCoro1.1 *: G*C coverage. BlobToolKit GC-coverage plot. Scaffolds are coloured by phylum. Circles are sized in proportion to scaffold length. Histograms show the distribution of scaffold length sum along each axis. An interactive version of this figure is available at https://blobtoolkit.genomehubs.org/view/idEupCoro1.1/dataset/CAMAOT01/blob.

**Figure 4.**
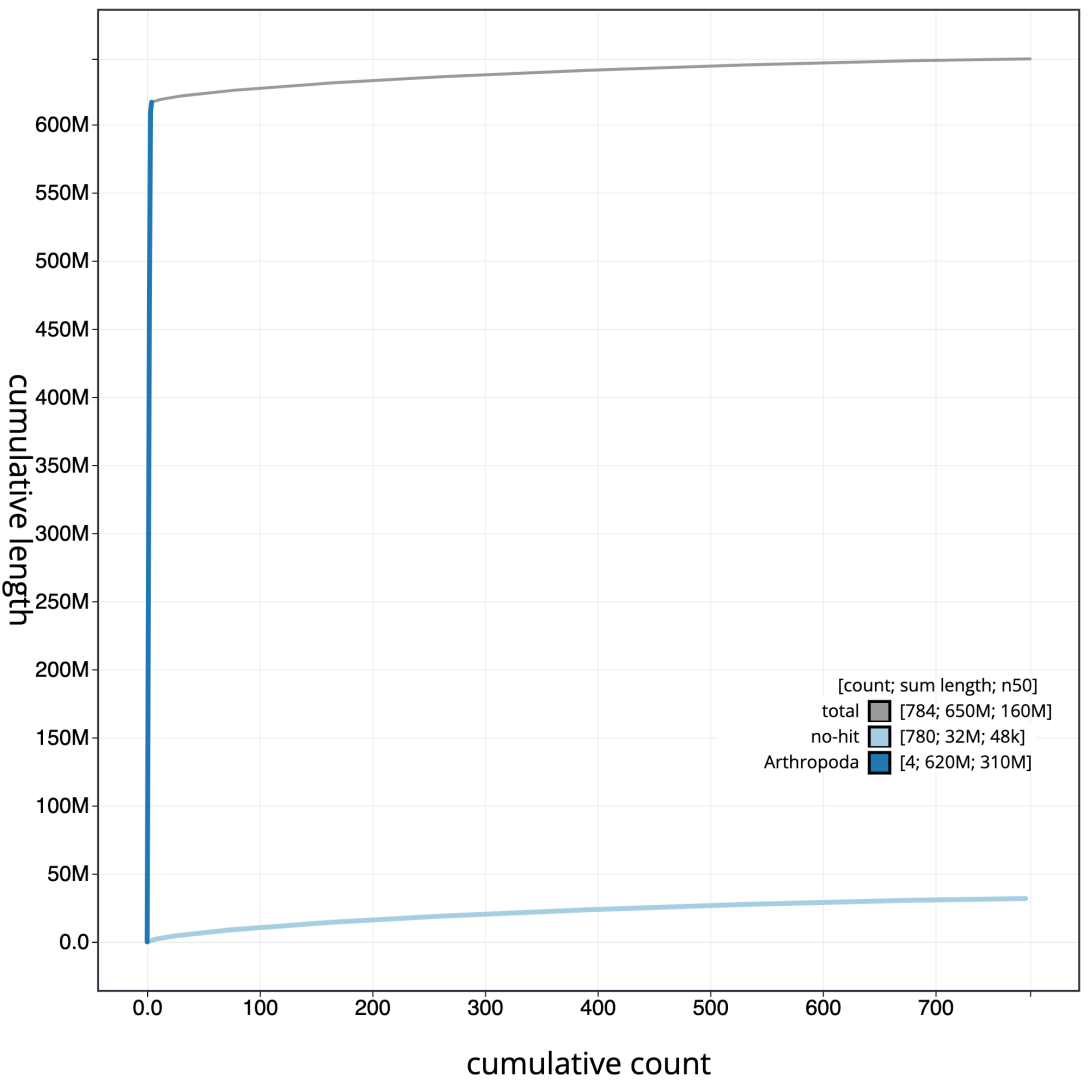
Genome assembly of *Eupeodes corollae,* idEupCoro1.1: cumulative sequence. BlobToolKit cumulative sequence plot. The grey line shows cumulative length for all scaffolds. Coloured lines show cumulative lengths of scaffolds assigned to each phylum using the buscogenes taxrule. An interactive version of this figure is available at https://blobtoolkit.genomehubs.org/view/idEupCoro1.1/dataset/CAMAOT01/cumulative.

**Figure 5.**
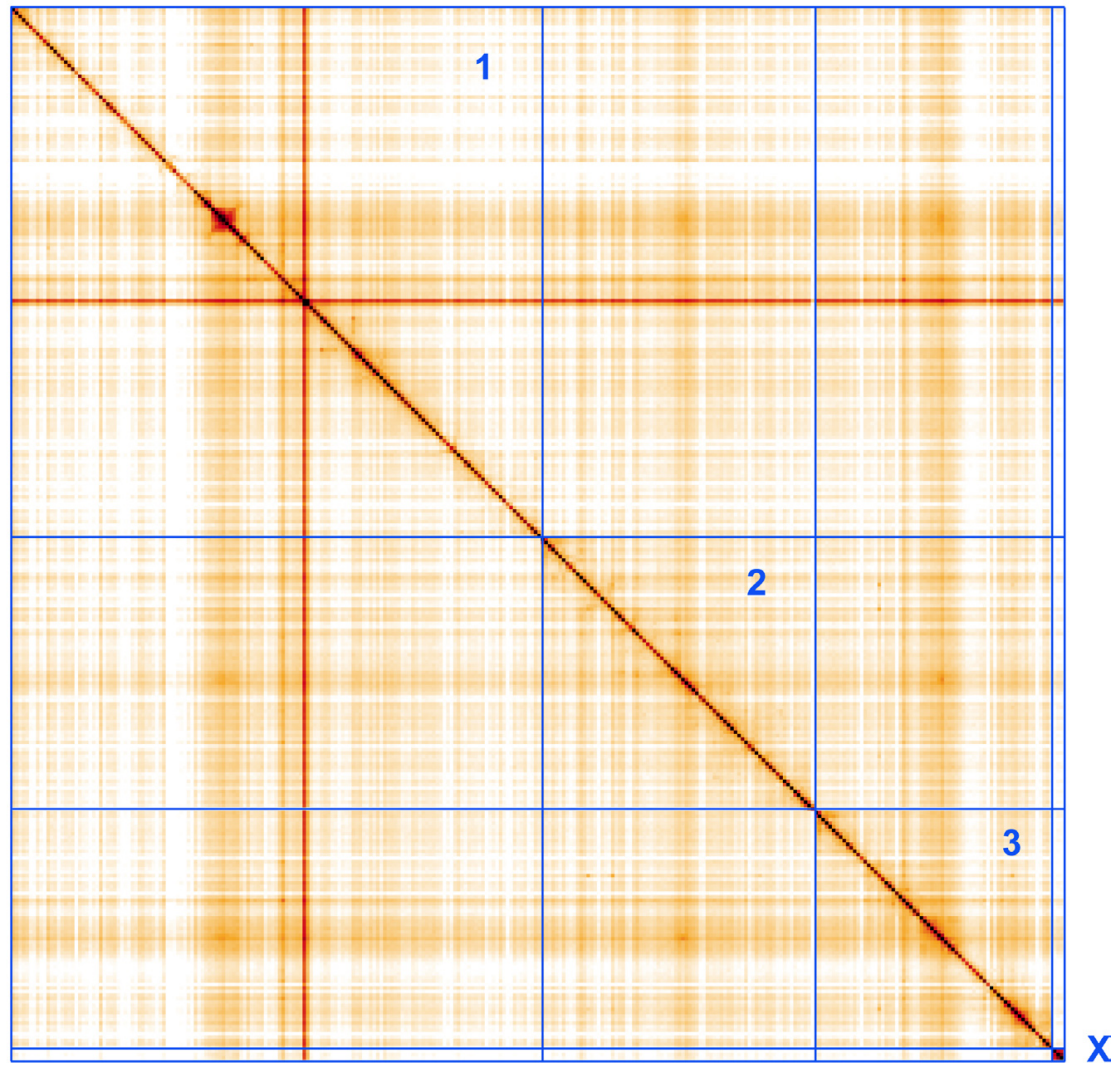
Genome assembly of *Eupeodes corollae,* idEupCoro1.1: Hi-C contact map. Hi-C contact map of the idEupCoro1.1 assembly, visualised using HiGlass. Chromosomes are shown in order of size from left to right and top to bottom. An interactive version of this figure may be viewed at https://genome-note-higlass.tol.sanger.ac.uk/l/?d=RLyscKYrT8GuIgKEvXVwiQ.

**Table 2.** Chromosomal pseudomolecules in the genome assembly of *Eupeodes corollae,* idEupCoro1.

INSDC accession	Chromosome	Size (Mb)	GC%
OX244023.1	1	311.19	34.3
OX244024.1	2	158.62	33.5
OX244025.1	3	139.49	33.4
OX244026.1	X	7.12	32
OX244027.1	MT	0.02	17.8
-	unplaced	31.77	35.3

## Methods

### Sample acquisition and nucleic acid extraction

Two *Eupeodes corollae* specimens (idEupCoro1 and idEupCoro2) were collected by netting in an urban garden in Luton, UK (latitude 51.89, longitude –0.39) on 26 April 2020 and 16 June 2020. The specimens were collected by Olga and Duncan Sivell (Natural History Museum) and were preserved on dry ice. Both specimens were identified by Duncan Sivell using Stubbs and Falk (2002) and Ball & Morris (2015).

DNA was extracted at the Tree of Life laboratory, Wellcome Sanger Institute (WSI). The idEupCoro1 sample was weighed and dissected on dry ice. Tissue was disrupted using a Nippi Powermasher fitted with a BioMasher pestle. High molecular weight (HMW) DNA was extracted using the Qiagen MagAttract HMW DNA extraction kit. Low molecular weight DNA was removed from a 20 ng aliquot of extracted DNA using the 0.8X AMpure XP purification kit prior to 10X Chromium sequencing; a minimum of 50 ng DNA was submitted for 10X sequencing. HMW DNA was sheared into an average fragment size of 12–20 kb in a Megaruptor 3 system with speed setting 30. Sheared DNA was purified by solid-phase reversible immobilisation using AMPure PB beads with a 1.8X ratio of beads to sample to remove the shorter fragments and concentrate the DNA sample. The concentration of the sheared and purified DNA was assessed using a Nanodrop spectrophotometer and Qubit Fluorometer and Qubit dsDNA High Sensitivity Assay kit. Fragment size distribution was evaluated by running the sample on the FemtoPulse system.

### Sequencing

Pacific Biosciences HiFi circular consensus and 10X Genomics read cloud DNA sequencing libraries were constructed according to the manufacturers’ instructions. DNA sequencing was performed by the Scientific Operations core at the WSI on Pacific Biosciences SEQUEL II (HiFi) and HiSeq X Ten (10X) instruments. Hi-C data were also generated from specimen idEupCoro2 using the Arima v2 kit and sequenced on the Illumina NovaSeq 6000 instrument.

### Genome assembly

Assembly was carried out with Hifiasm ( Cheng *et al.*, 2021) and haplotypic duplication was identified and removed with purge_dups ( Guan *et al.*, 2020). One round of polishing was performed by aligning 10X Genomics read data to the assembly with Long Ranger ALIGN, calling variants with FreeBayes ( Garrison & Marth, 2012). The assembly was then scaffolded with Hi-C data ( Rao *et al.*, 2014) using YaHS ( Zhou *et al.*, 2023). The assembly was checked for contamination and corrected using the gEVAL system ( Chow *et al.*, 2016) as described previously ( Howe *et al.*, 2021). Manual curation was performed using gEVAL, HiGlass ( Kerpedjiev *et al.*, 2018) and Pretext ( Harry, 2022). The mitochondrial genome was assembled using MitoHiFi ( Uliano-Silva *et al.*, 2022), which performed annotation using MitoFinder ( Allio *et al.*, 2020). The genome was analysed and BUSCO scores were generated within the BlobToolKit environment ( Challis *et al.*, 2020). Table 3 contains a list of all software tool versions used, where appropriate.

**Table 3.** Software tools and versions used.

Software tool	Version	Source
BlobToolKit	3.5.0	Challis *et al.*, 2020
FreeBayes	1.3.1-17- gaa2ace8	Garrison & Marth, 2012
gEVAL	N/A	Chow *et al.*, 2016
Hifiasm	0.16.1-r375	Cheng *et al.*, 2021
HiGlass	1.11.6	Kerpedjiev *et al.*, 2018
Long Ranger ALIGN	2.2.2	https://support.10xgenomics.com/ genome-exome/software/pipelines/ latest/advanced/other-pipelines
MitoHiFi	2	Uliano-Silva *et al.*, 2022
PretextView	0.2	Harry, 2022
purge_dups	1.2.3	Guan *et al.*, 2020
YaHS	yahs- 1.1.91eebc2	Zhou *et al.*, 2023

### Ethics and compliance issues

The materials that have contributed to this genome note have been supplied by a Darwin Tree of Life Partner. The submission of materials by a Darwin Tree of Life Partner is subject to the Darwin Tree of Life Project Sampling Code of Practice. By agreeing with and signing up to the Sampling Code of Practice, the Darwin Tree of Life Partner agrees they will meet the legal and ethical requirements and standards set out within this document in respect of all samples acquired for, and supplied to, the Darwin Tree of Life Project. All efforts are undertaken to minimise the suffering of animals used for sequencing. Each transfer of samples is further undertaken according to a Research Collaboration Agreement or Material Transfer Agreement entered into by the Darwin Tree of Life Partner, Genome Research Limited (operating as the Wellcome Sanger Institute), and in some circumstances other Darwin Tree of Life collaborators.

## Data Availability

European Nucleotide Archive: *Eupeodes corollae*. Accession number PRJEB53926; https://identifiers.org/ena.embl/PRJEB53926. ( Wellcome Sanger Institute, 2022) The genome sequence is released openly for reuse. The *Eupeodes corollae* genome sequencing initiative is part of the Darwin Tree of Life (DToL) project. All raw sequence data and the assembly have been deposited in INSDC databases. The genome will be annotated using available RNA-Seq data and presented through the Ensembl pipeline at the European Bioinformatics Institute. Raw data and assembly accession identifiers are reported in Table 1.

## Author information

Members of the Natural History Museum Genome Acquisition Lab are listed here: https://doi.org/10.5281/zenodo.4790043.

Members of the Darwin Tree of Life Barcoding collective are listed here: https://doi.org/10.5281/zenodo.4893703.

Members of the Wellcome Sanger Institute Tree of Life programme are listed here: https://doi.org/10.5281/zenodo.4783585.

Members of Wellcome Sanger Institute Scientific Operations: DNA Pipelines collective are listed here: https://doi.org/10.5281/zenodo.4790455.

Members of the Tree of Life Core Informatics collective are listed here: https://doi.org/10.5281/zenodo.5013541.

Members of the Darwin Tree of Life Consortium are listed here: https://doi.org/10.5281/zenodo.4783558.
