# Supplementary material for: The genome sequence of the Vagrant Hoverfly, Eupeodes corollae (Fabricius, 1794)
Source: Wellcome Open Res. 2023 Mar 8;8:112. [Version 1] doi: 10.12688/wellcomeopenres.19099.1 (PMC10556570; doi:10.12688/wellcomeopenres.19099.1)
Supplement: Peer review history

# Reviewer report: Reviewer response for version 1

**DOI:** 10.21956/wellcomeopenres.21175.r67747

**Published:** 2023-10-06

**Authors:** Rosario Nicoletti

**Affiliations:** 1 University of Naples Federico II, Portici, Italy

This article reports on the genome sequencing of a hoverfly species ( *E. corollae*) based on a specimen collected in the United Kingdom. It is concise and well written, and provides an adequate account on the relevant information concerning this insect. Although not absolutely necessary, an useful integration of the background section could derive by considering data reported in the papers by Pu et al. (2017) *Scientific Reports* *7*, 44300, and Falk et al. (2022) *Wellcome Open Research*, *7; *particularly, the latter concerns genome sequencing of a related species, also obtained within the Darwin Tree of Life project. A few minor corrections are required in the text, that is: using the abbreviated form ' *E. corollae*' in the final sentence of the background section; the word 'genome' not to be written in italics in the same sentence; writing ' *E. corollae*' in italics in the previous paragraph.

Are sufficient details of methods and materials provided to allow replication by others?

Yes

Is the rationale for creating the dataset(s) clearly described?

Yes

Are the datasets clearly presented in a useable and accessible format?

Yes

Are the protocols appropriate and is the work technically sound?

Yes

Reviewer Expertise:

Mycology, plant pathology, entomopathogens.

I confirm that I have read this submission and believe that I have an appropriate level of expertise to confirm that it is of an acceptable scientific standard.

## References

[1] : Mitochondrial genomes of the hoverflies Episyrphus balteatus and Eupeodes corollae (Diptera: Syrphidae), with a phylogenetic analysis of Muscomorpha. *Sci Rep* .2017;7: 10.1038/srep44300 44300 10.1038/srep44300 28276531PMC5343577

[2] : The genome sequence of the meadow field syrph, Eupeodes latifasciatus (Macquart, 1829). *Wellcome Open Res* .2022;7: 10.12688/wellcomeopenres.18113.1 253 10.12688/wellcomeopenres.18113.1 36938120PMC10015120

# Reviewer report: Reviewer response for version 1

**DOI:** 10.21956/wellcomeopenres.21175.r67740

**Published:** 2023-10-06

**Authors:** Jaakko L.O. Pohjoismäki

**Affiliations:** 1 Department of Environmental and Biological Sciences, University of Eastern Finland, Joensuu, Finland

Background: Maybe worth noting that the coloration among the species is variable and especially the females might be difficult to determine. Would be also informative to tell that there are 8 other Eupeodes species in the UK plus one, whose taxonomic status is uncertain. See the species list at: http://hoverfly.uk/

 Not much to comment on the genome sequencing, it nicely follows the DToL pipeline, quality standards and presentation.

 Methods: The sex of the specimens is not specified?

 Otherwise, I have the same complaint as I have had with other similar reports. Although the mitochondrial genome was retrieved the Cox1 DNA barcode has not been checked. I did this and it seems to confirm the species identity.

 DNA barcodes work quite poor for some Syrphid genera, but for this species the identify should be ok.

 I also noted that the mitochondrial genome produced here that is available in the GenBank has not been annotated and is in reverse orientation?

https://www.ncbi.nlm.nih.gov/nuccore/OX244027.1?report=genbank

Are sufficient details of methods and materials provided to allow replication by others?

Yes

Is the rationale for creating the dataset(s) clearly described?

Yes

Are the datasets clearly presented in a useable and accessible format?

Yes

Are the protocols appropriate and is the work technically sound?

Yes

Reviewer Expertise:

Mitochondrial DNA, molecular biology, genetics, taxonomy

I confirm that I have read this submission and believe that I have an appropriate level of expertise to confirm that it is of an acceptable scientific standard, however I have significant reservations, as outlined above.

# Reviewer report: Reviewer response for version 1

**DOI:** 10.21956/wellcomeopenres.21175.r67741

**Published:** 2023-10-05

**Authors:** Shigeyuki Koshikawa, Takumi Karasawa

**Affiliations:** 1 Graduate School of Environmental Science, Hokkaido University, Sapporo, Japan; 2 Graduate School of Environmental Science, Hokkaido University, Sapporo, Hokkaido Prefecture, Japan

This article reports a high-quality genome sequence of the Vagrant Hoverfly, Eupeodes corollae. The taxonomic position and ecology of this species are briefly presented. The methods used to obtain the genome sequence are explained in an appropriate amount of detail. We believe this article will promote an understanding of the nature of this species, regional biodiversity, and insect biology in general.

 We have a few minor comments below.

 The last e letter in the species name in the article title should be in italics.

 In the introduction, the authors wrote that a genome assembly of this species has been published before. We think the authors should describe how much better this genome assembly is compared to that earlier assembly.

 It is not clear what the 311M in the legend in the lower-left corner of Figure 2 indicates.

 It would be easier to understand if there was a diagram showing how much the size of the circle in Figure 3 corresponds to the scaffold length. Also, it is difficult to understand what the Y-axis in Figure 3, which says ERR9904177_cov, indicates.

 The blue letters indicating chromosomes 1-3 in Figure 5 cover the figure. It would be better to move them outside the figure.

Are sufficient details of methods and materials provided to allow replication by others?

Yes

Is the rationale for creating the dataset(s) clearly described?

Yes

Are the datasets clearly presented in a useable and accessible format?

Yes

Are the protocols appropriate and is the work technically sound?

Yes

Reviewer Expertise:

Evolutionary Developmental Biology

We confirm that we have read this submission and believe that we have an appropriate level of expertise to confirm that it is of an acceptable scientific standard.

# Reviewer report: Reviewer response for version 1

**DOI:** 10.21956/wellcomeopenres.21175.r63583

**Published:** 2023-08-08

**Authors:** Josephine Reinhardt

**Affiliations:** 1 State University of New York at Geneseo, Geneseo, NY, USA

This report is of the genome of the Vagrant hoverfly *Eupeodes corollae*, produced as part of the Darwin Tree of Life (DToL) consortium.  As such, it uses standardized methods and reports standardized results in line with genome reports of other arthropod species produced by the consortium. The brief introduction to the species and its interest to the scientific community and broader public appears appropriate. The approach DToL is taking with their genome reports has great benefits, because makes it a simple matter to compare genome assembly quality across taxa. The genome is of high quality based on the metrics presented. I also checked that the data were available as reported (they were).  

 As a reader that hasn't personally worked with DToL genomes, there are a few places that I think could be improved in terms of clarifying for a broader audience referencing these reports. 

 Figure 2 - a "pale grey spiral" is referenced - I think this is meant to be the small light grey blob in the middle. This does not appear to be a spiral here, but is more spiral-like in similar reports e.g. https://www.ncbi.nlm.nih.gov/pmc/articles/PMC10349268/figure/f2/ ). Someone only looking at this report without seeing others could have trouble interpreting. There are also light grey (white?) dashed lines that are barely visible in the figure especially over the orange area. What are these and could the color be adjusted for visibility?  

 Figure 3: The Y-axis label is "ERR9904177_cov" and this ID does not appear anywhere else in the report so perhaps should be changed. This appears to be sequencing coverage, but multiple types of sequencing were done - both PacBioHiFi (34x) and 10X Genomics (34x). Which coverage is presented here? 

 Methods - the amount of DNA that was submitted for 10X sequencing (50ng) is mentioned but not the amount of DNA that was used in producing the HiFi and HiC libraries. Readers attempting to sequence genomes using multiple technologies from single small bodied individuals might be interested to know this information for planning their studies.

Are sufficient details of methods and materials provided to allow replication by others?

Yes

Is the rationale for creating the dataset(s) clearly described?

Yes

Are the datasets clearly presented in a useable and accessible format?

Yes

Are the protocols appropriate and is the work technically sound?

Yes

Reviewer Expertise:

Evolution, Genomics

I confirm that I have read this submission and believe that I have an appropriate level of expertise to confirm that it is of an acceptable scientific standard.
